# Machine learning-based ground motion simulation for seismic hazard assessment of critical water infrastructure in Azerbaijan (Case study: Major Shamkir water reservoir)

**DOI:** 10.1371/journal.pone.0344984

**Published:** 2026-04-01

**Authors:** Tural Babayev, Gulam Babayev, Emil Bayramov, Sonny Irawan, Jessica Neafie, Saida Aliyeva

**Affiliations:** 1 Department of Seismology and Seismic Hazard Assessment, Institute of Geology and Geophysics, Ministry of Science and Education, Baku, Azerbaijan; 2 Department of Geological Sciences, School of Mining and Geosciences, Nazarbayev University, Astana, Kazakhstan; 3 Department of Petroleum Engineering, School of Mining and Geosciences, Nazarbayev University, Astana, Kazakhstan,; 4 Department of Political Science and International Relations, School of Sciences and Humanities, Nazarbayev University, Astana, Kazakhstan; 5 School of Agricultural and Food Sciences, ADA University, Baku, Azerbaijan; Khalifa University of Science and Technology, UNITED ARAB EMIRATES

## Abstract

This study focuses on simulating ground motion for the Shamkir Water Reservoir area in Azerbaijan using machine learning algorithms to enhance regional seismic hazard assessments. Given the reservoir’s location in a seismically active zone, potential earthquake-induced impacts on dam infrastructure pose critical safety concerns. A synthetic ground motion database comprising 3,013 records was developed using SeismoArtif software, utilizing earthquake magnitude, hypocentral distance, average shear-wave velocity (V_S30_) and site class as primary features. Four supervised machine learning models—Artificial Neural Network (ANN), Random Forest (RF), Support Vector Machine (SVM), and Extreme Gradient Boosting (XGBoost)—were developed to predict Peak Ground Acceleration (PGA). A dual-layered performance evaluation was conducted, comparing the models against each other and against the traditional A&K-1979 Ground Motion Prediction Equation (GMPE). Results demonstrate that while all machine learning models are highly applicable and physically consistent, the traditional GMPE fails significantly, yielding R^2^ of −6.87 and MAE of 284.88 Gals. Within the machine learning cohort, the Random Forest model achieved the highest training scores (R^2^: 0.8598), yet the Artificial Neural Network (ANN) emerged as the optimal architecture due to its superior generalization and stability. The ANN led the decisive testing phase with an R^2^ of 0.8437, an RMSE of 46.00 Gals, and the lowest systematic bias (−1.76 Gals) across all subsets. These findings underscore the robustness of data-driven approaches over fixed-coefficient empirical relationships, specifically highlighting the ANN’s capability to provide unbiased, high-fidelity ground motion estimations for critical infrastructure risk-informed decision-making in Azerbaijan.

## Introduction

Earthquakes have long been among the most devastating natural hazards, responsible for substantial human casualties and extensive economic damage throughout history. Their impact is particularly severe in tectonically active regions, where recurrent seismic events pose an ongoing threat to communities and infrastructure. In response to this persistent danger, seismic hazard assessment has emerged as a critical scientific discipline aimed at quantifying the expected ground motion generated by potential earthquakes. This assessment forms the foundation for earthquake-resistant engineering, informs risk-sensitive land-use planning, and underpins disaster risk reduction strategies. Any seismic hazard analysis fundamentally requires the consideration of four key parameters: the earthquake magnitude, the distance between the earthquake source and the site of interest, the local site conditions, and the expected ground motion corresponding to the given magnitude, distance and site characteristics [1].

Ground motion modeling plays a central role in seismic hazard assessment by providing estimates of the expected ground motion levels from potential earthquakes. Traditionally, this is achieved through the development and application of Ground Motion Prediction Equations (GMPEs), which are empirical models derived from recorded earthquake data specific to the study region. These equations relate ground motion intensity to variables such as magnitude, distance, and site conditions. In recent years, data-driven methodologies—particularly machine-learning-based models—have gained increasing attention as alternatives or complements to conventional ground motion prediction equations (GMPEs), owing to their enhanced predictive capability and flexibility in capturing complex, region-specific seismic characteristics [2].

Ground motion estimations are commonly expressed in terms of ground motion parameters such as peak ground acceleration (PGA) and peak ground velocity (PGV). Data-driven approaches, including machine learning (ML)-based models, rely heavily on the availability and quality of seismic datasets to ensure accurate predictions of these parameters [3]. Similarly, Khosravikia and Clayton [4] emphasize that the accuracy of ML-based models improves significantly with the presence of large and high-quality datasets.

Ground Motion Prediction Equations (GMPEs) remain widely used empirical tools in seismic hazard analysis and are typically developed for specific regions. For instance, several GMPEs have been proposed for Türkiye by Bindi et al. [5], Akkar and Cagnan [6], Kale et al. [7], Gülkan and Kalkan [8], Akinci et al. [9], Akyol and Karagöz [10], Kayabalı and Beyaz [11], Gençoğlu and Sayıl [12].

Recent research has compared the performance of ML-based ground motion models (GMMs) with traditional GMPEs, often showing promising results [13]. Moreover, comparative studies have been conducted to evaluate different ML algorithms for ground motion prediction tasks [4]. These algorithms have been applied across various regions, including Türkiye [14], Europe [15], Taiwan [16], Japan [17,18], Poland [19], and the United States [20]. For example, Khosravikia and Clayton [4] reported that traditional GMPEs yielded the lowest predictive performance compared with ANN, RF, and SVM models when evaluated using 4,528 ground motion records from 209 seismic stations in Texas, Oklahoma, and Kansas.

Among the commonly used ML algorithms in this field are Artificial Neural Networks (ANN), Random Forest (RF), Support Vector Machines (SVM) and Extreme Gradient Boosting (XGBoost). These methods are particularly effective in capturing nonlinear relationships and feature interactions within complex seismic datasets [3,4,21]. In this context, Babayev et al. [2] developed an ANN-based, data-driven ground motion model for Azerbaijan. Nevertheless, the authors highlighted inherent limitations associated with the quality and completeness of the available observational dataset and consequently advocated the use of synthetic ground motion simulations as a practical means of improving the robustness and reliability of seismic hazard assessments in the region.

Synthetic dataset generation is widely recognized as an effective solution to compensate for the scarcity of real ground motion data, especially in regions with limited seismic recordings. Such datasets are extensively employed in data-driven ground motion modeling, particularly when the available empirical data are insufficient for robust training of machine learning algorithms. Various techniques exist for generating synthetic datasets, among which stochastic methods are commonly used. Boore [22] laid the foundation for stochastic simulation by integrating the theoretical models of Aki [23], Brune [24], Hanks and McGuire [25]. Beresnev and Atkinson [26] later introduced finite-fault effects into the framework, and Motazedian and Atkinson [27] further refined the approach by addressing several of its limitations, resulting in the stochastic finite-fault method.

This methodology has seen wide application in recent years. For example, Karimzadeh et al. [28] applied the Motazedian and Atkinson [27] approach to simulate ground motions in Türkiye and subsequently developed an ANN-based ground motion model using the generated dataset. Similarly, Temiz et al. [29] created an ANN-based GMM for the North Tabriz Fault using a synthetic dataset composed of 206,382 simulated acceleration time series, generated via the EXSIM platform using the same stochastic method. Beyond model development, ground motion simulations have been used to reconstruct and analyze historical earthquakes. Tanırcan and Yelkenci-Necmioğlu [30] simulated the 2017 Bodrum-Kos earthquake, while Can et al. [31] modeled the 2002 Çay earthquake in Türkiye. Numerous other studies have used stochastic simulations to address the lack of strong-motion recordings for significant earthquakes, including the 1999 Düzce (Mw 7.1), 1939 and 1992 Erzincan (Mw 7.8 and 6.6), 2020 Elazığ (Mw 6.8), and 2023 Kahramanmaraş (Mw 7.8) earthquakes [32–37]. In addition, recent works have explored validation of synthetic records from both seismological and engineering perspectives [38–41]. Collectively, these findings support the emerging consensus that synthetic ground motion simulations constitute a powerful and effective approach for overcoming the constraints imposed by sparse instrumental records, particularly for large-magnitude earthquakes that are poorly represented in empirical datasets [2].

In this study, we aim to develop ML-based GMMs—specifically using ANN, RF, SVM and XGBoost—to estimate PGA for the Shamkir Water Reservoir region, located in the Middle Kur Depression of Azerbaijan. Given the limited availability of strong-motion data in the region, synthetic acceleration time series are generated using the contemporary SeismoArtif software developed by SeismoSoft. The resulting dataset is used to train the proposed GMMs, which take magnitude, hypocentral distance, V_S30_ (shear-wave velocity in the upper 30 meters) and site classification as input parameters. Thus, the research is conducted in two main stages: (i) data generation using the SeismoArtif software and (ii) development of GMMs using ML algorithms trained on the generated dataset. The trained models are made openly accessible through a GitHub repository, providing a user-friendly framework for estimating PGA based on user-defined seismic parameters. In addition, a Python script is provided to load the trained models and the associated source-to-site parameters, read the input variables—including magnitude, hypocentral distance, V_S30_, and site class—and predict PGA using the selected model.

## Study area

**Fig 1:**
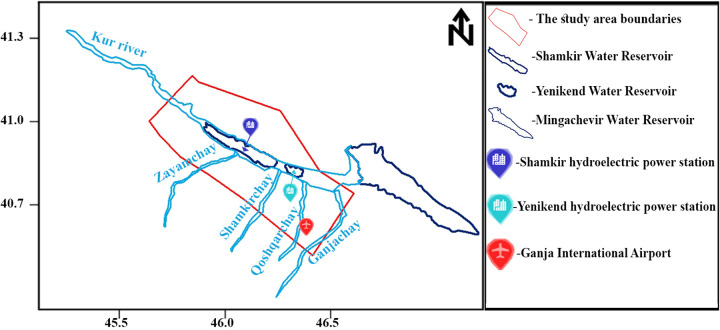
Map of the study area. The red polygon indicates the study boundaries.

This study focuses on the Shamkir Reservoir and its surrounding areas, located within the Shamkir region of the Republic of Azerbaijan. The selected study area encompasses the administrative regions of Shamkir, Ganja, Samukh and Tovuz. This region includes several strategically important infrastructures, such as the Shamkir Reservoir, Shamkir Hydroelectric Power Station, Yenikend Reservoir, Yenikend Hydroelectric Power Station and Ganja International Airport. The total area under study covers approximately 2,608.674 km² [42]. The geographic extent of the study area is delineated by a red polygon in Fig 1.

Geologically, the region lies within the Kur intermontane mega-zone, a major structural Depression forming part of the Intra-Caucasian depression system. This zone, which extends approximately 450 km from the Dzirul Protrusion in Georgia to the South Caspian Basin, lies between the Greater and Lesser Caucasus mountain-fold systems and belongs to the South Caucasus microplate [43,44]. The study area specifically falls within the Middle Kur depression and is characterized by a gently sloping foothill plain that descends from south to north.

The northern part of the study area features weakly consolidated sediments and volcanics from the Pliocene and Quaternary periods [45–47], while the southern section is dominated by Upper Pliocene Aghchagil sediments composed of clastic conglomerates, sandstones, silty claystones, clayey siltstones and volcanic ash [44,48].

**Fig 2:**
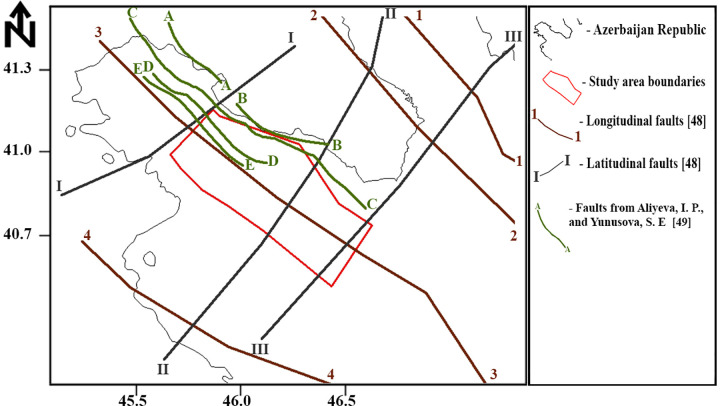
Fault map of the study area.

A prominent geological structure within the study area is the Kur Thrust Fault, which runs longitudinally through the region. This fault is particularly significant as it intersects both the Shamkir and Mingachevir reservoirs and hydroelectric power stations along the Kur River [44]. The fault system within the study area is illustrated in Fig 2. The fault system is adapted from [49,50].

**Fig 3:**
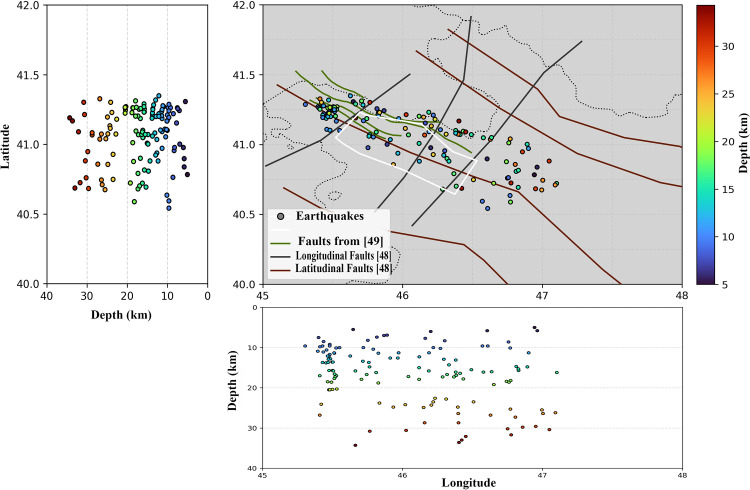
Seismicity-based identification of active faults.

To identify potential seismic sources, a catalog-based seismicity analysis was conducted using data from the International Seismological Centre [51] covering the years 1974–2022. This analysis allowed for the delineation of active faults and the estimation of their depths (Fig 3).

**Fig 4:**
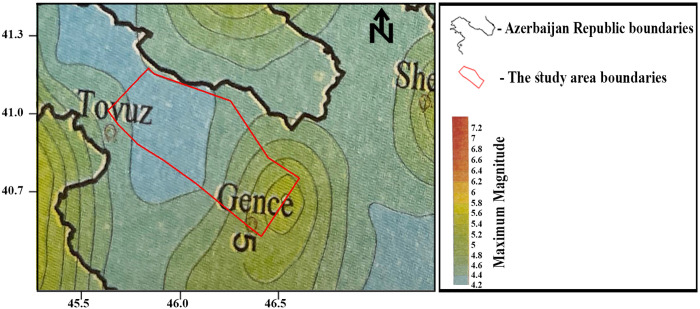
The maximum magnitude distribution of the study area. Adapted from [52].

The regional seismicity, in terms of the maximum magnitude distribution, was analyzed by Kadirov et al. [52] based on a seismicity catalog covering the 20^th^ and early 21^st^ centuries. Fig 4 presents the maximum magnitude distribution, with the study area adapted. The map indicates that the maximum expected magnitude within the study area ranges from 4.2 to 5.8. Accordingly, for the generation of synthetic accelerograms in this study, this magnitude range is considered with an increment of 0.2, following the approach adopted in [52]. Consequently, synthetic accelerograms are generated for magnitudes 4.2, 4.4, …, 5.6, and 5.8.

**Fig 5:**
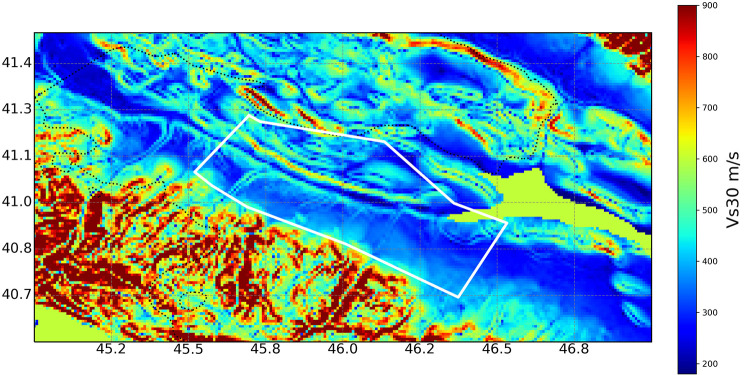
V_S30_ map of the study area. The white polygon indicates the study boundaries.

To generate synthetic accelerograms, site effects must be characterized. To incorporate site effects, a VS30 map was generated for the study area using global gridded data from the United States Geological Survey [53], processed using Python [54,55]. Fig 5 illustrates the V_S30_ values for the study area.

Site classification must be incorporated during synthetic data generation to properly account for site-specific amplification or attenuation effects. AzDTN [56], the national building code of Azerbaijan, defines four site classes based on V_S30_ ranges. Table 1 summarizes the site classifications adopted in Azerbaijan according to the corresponding V_S30_ intervals.

**Table 1:** Site classification in AzDTN based on V_S30_ values.

Site Class	Description	V_S30_ Range (m/s)
I	Hard soil/rock	V_S30_ > 800
II	Medium-dense soil	360 ≤ V_S30_ ≤ 800
III	Soft to medium soil	180 ≤ V_S30_ < 360
IV	Very soft soil	V_S30_ < 180

**Fig 6:**
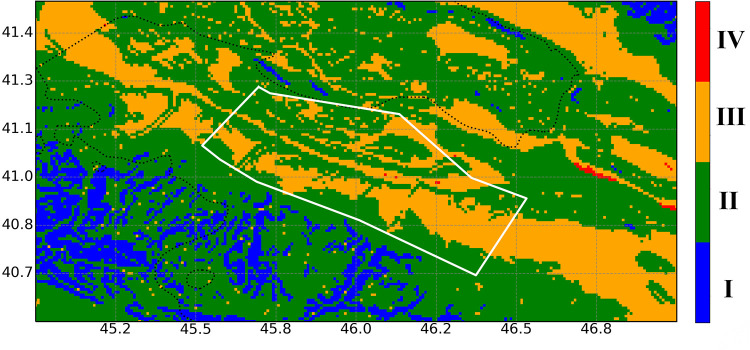
The site classification map of the study area. A negligible extent of Site Class IV is observed and therefore excluded from the synthetic dataset generation.

Based on this classification, the site class distribution map of the study area is presented in Fig 6.

As shown in Fig 6, the study area is characterized by Site Classes II and III, corresponding to medium-dense and soft-to-medium soil conditions. The amplification factors are 1.0 for Site Class II and 1.2 for Site Class III. These values indicate the absence of significant attenuation of seismic waves within the study area, underscoring the importance of conducting a detailed seismic hazard assessment. Furthermore, the presence of critical infrastructure—such as water reservoirs, hydroelectric power plants, and an international airport—necessitates heightened sensitivity to potential seismic vulnerabilities (Fig 1).

## Materials and methods

### Synthetic accelerogram generation methods of SeismoArtif

In this study, synthetic accelerograms are generated using SeismoArtif, a software tool developed by SeismoSoft. The program provides four distinct methods for accelerogram generation and modification:

- Synthetic Accelerogram Generation & Adjustment,
- Artificial Accelerogram Generation,
- Artificial Accelerogram Generation & Adjustment and
- Real Accelerogram Adjustment.

**Fig 7:**
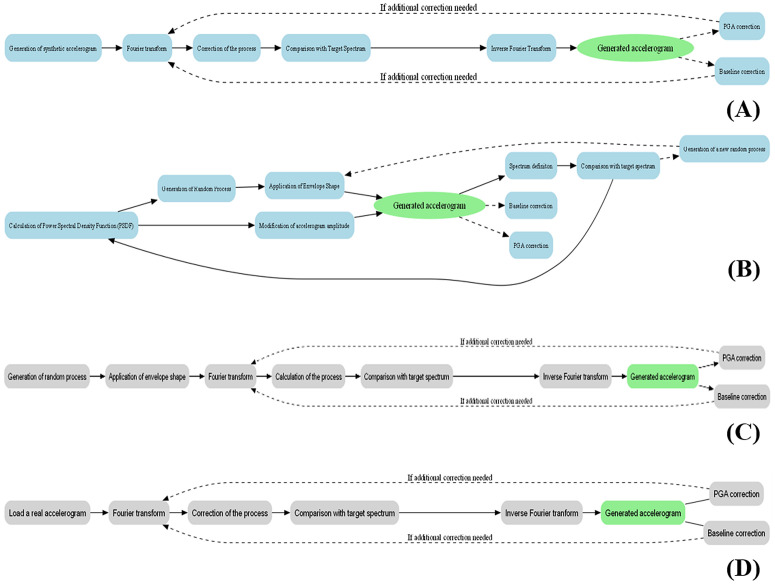
Schematic representation of the logical sequence of the four accelerogram generation methods implemented in the Seismoartif software. (a) “Synthetic accelerogram generation & adjustment” method. (b) “Artificial accelerogram generation” method. (c) “Artificial accelerogram generation & adjustment” method. (d) “Real accelerogram adjustment” method.

The method employed in this research is the “Synthetic Accelerogram Generation & Adjustment” approach, which is based on the methodology proposed by Halldorsson and Papageorgiou [57]. This technique produces synthetic accelerograms that are compatible with a given target response spectrum, which serves as the reference for spectral shaping. Target spectra can be derived from real earthquake recordings or from standard design spectra defined in seismic building codes. The overall procedure of this method is illustrated in Fig 7a.

Another method available in SeismoArtif is “Artificial Accelerogram Generation”, which is grounded in the assumption that any periodic signal can be represented as a sum of sinusoidal functions. In this approach, phase angles are assigned arbitrarily, while the amplitudes are computed using the power spectral density function (PSD) as formulated by Gasparini and Vanmarcke [58]. The logical workflow of this technique is depicted in Fig 7b.

In essence, this method synthesizes artificial accelerograms by superimposing a series of modulated sine waves. The frequency content and energy distribution of the resulting ground motion are governed by the PSD function, as expressed in Equation (1):


$$a(t)=\;q(t)\sum_{i=\text{1}}^{N}A_{i}\;sin(w_{i}t+\Phi_{i}),\;$$ (1)

where q(t) is the deterministic envelope function, $A_{i}$ is the amplitude of the sine wave, $w_{i}$ is the frequency of the sine wave and $\Phi_{i}$ is the phase of the sine wave.

An envelope function is a mathematical expression that defines the time-dependent upper bound of a wave’s amplitude. Various types of envelope functions have been proposed in the literature. Among the most commonly referenced are:

- The exponential envelope function, introduced by Liu [59], as shown in Equation (2),
- The partial envelope function, developed by Jennings et al. [60], shown in Equation (3) and
- The gamma envelope function, proposed by Saragoni and Hart [61], presented in Equation (4).

$$q(t)=\;a_{\text{0}}\left(e^{-\alpha t}-e^{-\beta t}\right),$$ (2)

where q(t) is the exponential envelope function proposed by Liu [59] and α and β are the constants of the envelope function.


$$q(t)=\;\left\{ \begin{array}{c} @l{\left(\frac{t}{T_{\text{1}}}\right)}^{\text{2}}\;\text{0}\leq t\leq \;T_{\text{1}}\;\\ \text{1}.\text{0}\;{\;T}_{\text{1}}\leq t\leq \;T_{\text{2}}\\ e^{-\alpha (t-T_{\text{2}})}\;\;T_{\text{2}}\leq t\leq \;T_{d} \end{array},\right.\;$$ (3)

where q(t) is the partial envelope function proposed by Jennings et al. [60], $T_{\text{1}}$ is the earthquake onset time, $T_{\text{2}}$ is the earthquake termination time, $T_{d}$ is the total earthquake duration, and α is the model parameter.


$$q\;(t)=\;\alpha_{\text{1}}t^{\alpha_{\text{2}}-\text{1}}e^{-\alpha_{\text{3}}t},\;t\geq \text{0},$$ (4)

where q(t) is the gamma envelope function proposed by Saragoni and Hart [61], and $\alpha_{\text{1}}$, $\alpha_{\text{2}}$, $\alpha_{\text{3}}$ are the model parameters.

The third method available in Seismoartif is the “Artificial Accelerogram Generation & Adjustment” approach. Similar to the previous methods, it generates accelerograms that match a specified target spectrum. In this approach, adjustments are applied in both the frequency and time domains using Fourier transform techniques. Corrections to the random process components are made in the frequency domain, while envelope functions are employed to shape the time-domain characteristics of the signal. The logical workflow of this method is illustrated in Fig 7c.

The final method offered by Seismoartif is the “Real Accelerogram Adjustment” technique. In this approach, synthetic accelerograms are generated by modifying real earthquake records. Specifically, the frequency content of the selected real accelerogram is adjusted to conform to the target spectrum. The procedural steps of this method are shown schematically in Fig 7d.

### Generation of the synthetic database

As previously noted, the synthetic earthquake accelerograms used in this study are generated using the Synthetic Accelerogram Generation & Adjustment method, developed based on the approach proposed by Halldorsson and Papageorgiou [57]. The procedure for generating synthetic accelerograms with this method involves the following steps: (i) selecting the target response spectrum, (ii) inputting earthquake source parameters, (iii) defining site-specific soil conditions to account for local ground effects and (iv) generating the accelerogram based on the specified parameters in accordance with the target spectrum.

Selection of the Target Spectrum. As previously described, the target spectrum serves as the reference for generating synthetic accelerograms. Generally, this spectrum can be derived from two main sources: standardized building codes or recorded earthquake ground motions. SeismoArtif software provides three options for defining the target spectrum: (i) selecting from preloaded building code spectra (e.g., Eurocode 8), (ii) uploading acceleration time series data from a recorded earthquake, or (iii) manually importing user-defined spectra, such as national building codes not included in the software’s database.

**Fig 8:**
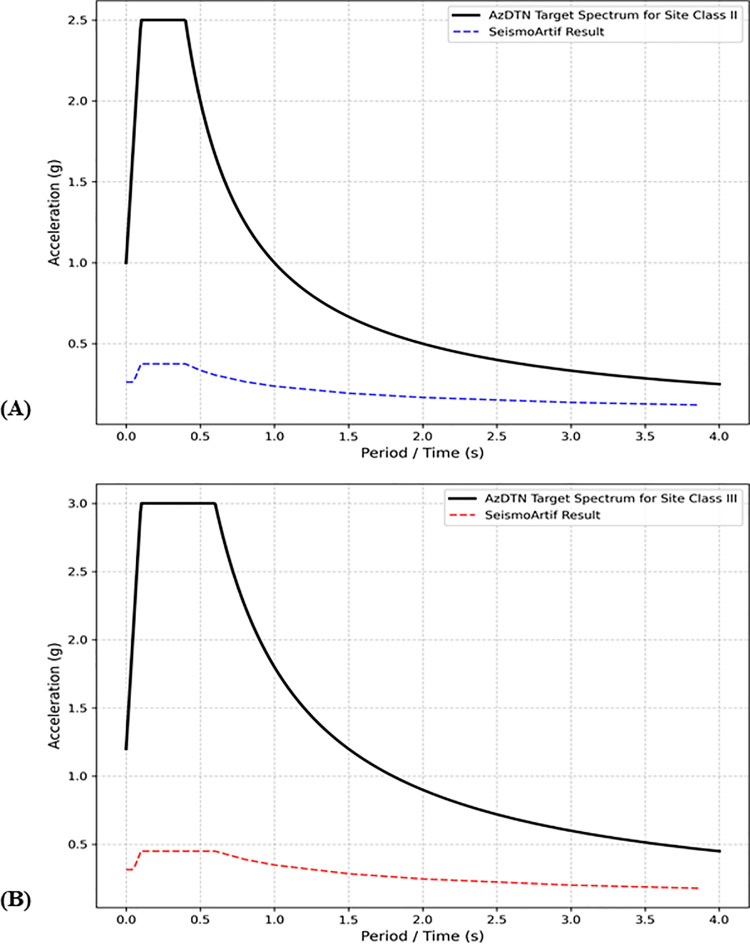
Acceleration spectrum functions defined by AzDTN for the site classes. (a) II and (b) III.

Since the Azerbaijani building code AzDTN [56] is not pre-integrated into the SeismoArtif software, we initially attempted to apply the third option, which involves uploading AzDTN acceleration time series specified for the site classes present in the study area. However, a technical issue in SeismoArtif emerged at this stage of the workflow. Specifically, the software does not correctly process target spectra derived from manually uploaded building codes, whereas it functions properly when preloaded codes are selected. To illustrate this issue, Fig 8 compares the original target spectra for Site Classes II and III defined by AzDTN—plotted by the authors using the Python Matplotlib library [62]—with the spectra generated by SeismoArtif.

As shown in Fig 8, the functional shapes of the spectra are consistent between the original AzDTN versions and those proposed by SeismoArtif. However, SeismoArtif yields substantially lower acceleration amplitudes, which results in the generation of synthetic accelerograms that have a negligible impact on the study area in terms of seismic hazard.

Consequently, one of the preloaded code options was adopted. Among the available alternatives, Eurocode 8 (EC8) represents the closest equivalent to AzDTN. Both AzDTN and EC8 define identical site classification schemes, in which Site Classes I, II, III, and IV in AzDTN correspond to Site Classes A, B, C, and D in EC8, respectively, with the same V_S30_ range groupings [63]. Moreover, EC8 is one of the reference codes cited by AzDTN during its adoption process [56]. Nevertheless, EC8 was not applied directly using its default settings. Instead, several parameters were adjusted to better reflect local conditions. For Site Class II, the ground type was selected as B, the reference peak ground acceleration was set to 0.125 g in accordance with AzDTN provisions [56], and the importance class was defined as Class IV. Importance classes in EC8 are associated with scaling factors of 0.8, 1.0, 1.2, and 1.4 for Classes I, II, III, and IV, respectively. While Class II represents the default and most commonly used category, the software applies the corresponding scaling factor by multiplying it with the specified reference acceleration. Importance Class I corresponds to structures of low importance, such as agricultural buildings, sheds, and minor storage facilities. Class II represents standard residential and commercial buildings. Class III includes structures associated with elevated risk, such as schools, assembly halls, and cultural institutions. Class IV accounts for critical infrastructure, including hospitals, fire stations, power plants, and large dams. Given that the study area contains two water reservoirs, two hydroelectric power plants, and an international airport, Importance Class IV was selected to appropriately account for the critical nature of the exposed assets. For Site Class III, the target spectrum was defined using Ground Type C and a reference acceleration of 0.250 g, consistent with AzDTN requirements [56].

Next, the earthquake and soil parameters were defined in the software. Earthquakes with hypocentral distances ranging from 1 to 35 km were classified as near-field events, whereas those occurring at greater distances were considered far-field. Regarding soil parameters, the V_S30_ value was assigned to specific locations within the study area where the simulations were performed. These locations corresponded to the corner points of the 10 km × 10 km grid cells into which the study area was divided.

This approach enables the generation of synthetic earthquakes tailored to the seismic and geotechnical characteristics of any given location. In this study, two site classes, a magnitude range of 4.2–5.8 with a step of 0.2, and a hypocentral distance range of 1–140 km with a step of 5 km were considered for the construction of the synthetic database. For each combination of parameters, the software generated seven synthetic accelerograms, resulting in a total of 3,654 (2 × 9 × 29 × 7) synthetic acceleration time series. Each accelerogram was labeled according to the format: class–magnitude–distance–version. For example, the first accelerogram (2-4.2-1-1) corresponds to Site Class II (2), magnitude 4.2, hypocentral distance of 1 km, and version 1. The last element in the database (3-5.8-140-7) corresponds to Site Class III (3), magnitude 5.8, hypocentral distance of 140 km, and version 7. All accelerograms were checked for convergence to the target spectrum. A total of 641 accelerograms that failed to converge were removed, leaving 3,013 validated synthetic accelerograms in the final database.

### Validation and characterization of the synthetic database

A synthetic database was compiled using the 3,013 synthetic earthquake acceleration time series.

**Fig 9:**
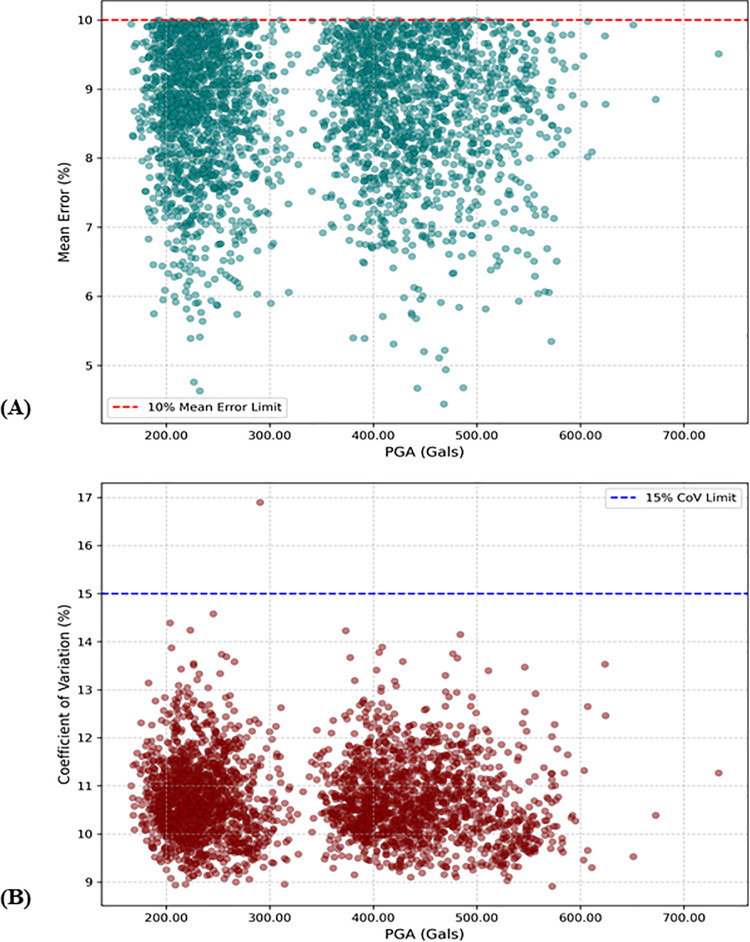
The validation plots of the synthetic acceleration time series. (a) Mean Error. (b) Coefficient of Variation.

To validate the synthetic accelerograms, SeismoArtif evaluates their convergence to the target spectrum using the Mean Error (ME) and the Coefficient of Variation (CoV). Convergence is considered achieved if ME is less than 10%, which is the software’s default threshold. ME quantifies the average difference between the target spectrum and the synthetic spectrum across all periods, reflecting the overall energy accuracy of the accelerogram. High ME indicates that PGA may under- or overestimate the shaking for the corresponding site class. CoV serves as a precision metric, describing the variability of the error across different periods. It is calculated as the standard deviation of the errors divided by ME. Low CoV (<15%) indicates a close match across all frequencies, producing a synthetic waveform that reliably represents the target spectrum. High CoV (>25%) indicates a “ragged” match, where errors at specific periods may deviate substantially despite an acceptable ME. For example, ME = 9% with high CoV may correspond to +30% error at T = 0.2 s and –20% at T = 1.0 s. From the ML training perspective, high CoV introduces aleatory variability (noise), which can impair model training by obscuring the underlying GMM trend. Values of CoV between 15% and 25% are considered tolerable for a dataset of this size. Fig 9 presents the ME and CoV values for the 3,013 converged synthetic accelerograms.

For the validated database, ME ranges from 4.44% to 10%, while CoV varies between 8.91% and 14.58%. Only one element (2-5.4-25-5) exhibits a CoV of 16.9%, which falls within the intermediate range and is considered acceptable. This element corresponds to the fifth version of the accelerogram for the given parameters: a hypocentral distance of 25 km, magnitude 5.4, and Site Class II, with an ME of 7.91% (Fig 9).

**Fig 10:**
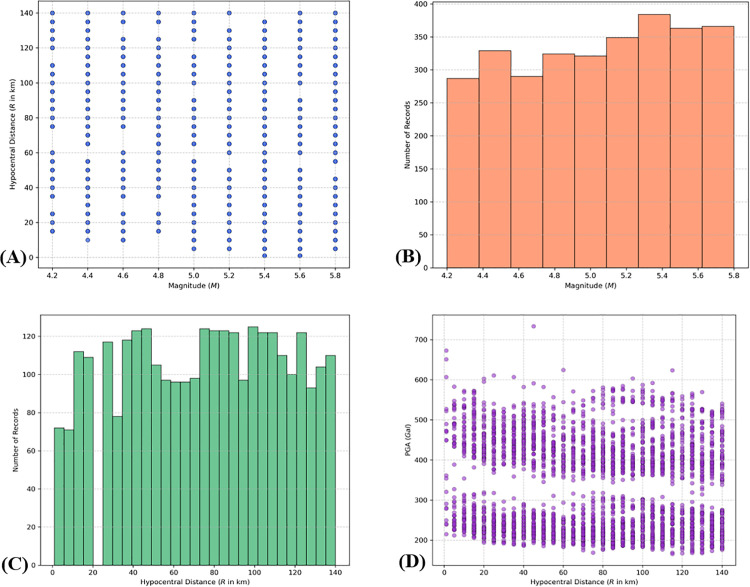
Distributions of the synthetic earthquake dataset. (a) Magnitude variation with respect to hypocentral distance. (b) Number of synthetic earthquakes by magnitude. (c) Number of synthetic earthquakes by hypocentral distance. (d) Hypocentral distribution of PGA values.

The characteristics of the validated synthetic database are further illustrated in Fig 10, which presents the distributions of various parameters across the dataset.

The statistical distributions and inter-parameter relationships of the expanded synthetic earthquake dataset (N = 3,013) are illustrated in Fig 10. Unlike stochastic real-world catalogs, this dataset was developed using a systematic grid-sampling approach to ensure comprehensive coverage across the feature space, which is essential for the stable convergence of ML algorithms. Fig 10a displays the distribution of magnitude (M) against hypocentral distance (r). The plot reveals a perfectly uniform grid, indicating that every magnitude increment (4.2 to 5.8) was paired with the full range of hypocentral distances (1–140 km). This “full-factorial” design eliminates the “gap” issues often found in empirical datasets (where large-magnitude events at short distances are rare), providing the ML models with a balanced training environment. The histograms in Figs 10b and 10c further confirm the balanced nature of the catalog. The magnitude distribution (Fig 10b) shows approximately equal representation across the nine discrete bins, while the distance distribution (Fig 10c) maintains a near-uniform density, with slight fluctuations resulting from the specific 5-km stepping logic. This uniformity ensures that the subsequent GMMs are not biased toward a specific earthquake scenario, thereby enhancing their predictive reliability across the entire studied range. A critical observation is found in Fig 10d, which depicts the variation of PGA with respect to hypocentral distance. The data exhibits two distinct “bands” of PGA values. This stratification is a direct result of the binary site conditions (Site Class = 2 and 3) incorporated into the simulation. The upper band represents the amplified response of the softer site class (Class III), while the lower band corresponds to the stiffer site condition (Class II). While the overall trend shows the expected physical attenuation of PGA with increasing distance, the vertical spread at any given distance reflects the influence of magnitude scaling and the seven synthetic variations generated for each scenario. Overall, the synthetic PGA values range from approximately 165–734 Gals, providing a clear picture of the target dataset’s scale, with a mean PGA of 338 Gals. The dataset offers a wider range of seismic parameters and a higher degree of resolution. This increased variability is expected significant generalization capabilities of the ML-GMMs. By providing a clear distinction between site classes and a dense sampling of the M − R space, the dataset provides a rigorous foundation for the ANN, XGBoost, RF, and SVM algorithms to learn the complex, nonlinear physics of ground motion attenuation without the constraints of traditional fixed-coefficient regression models.

### The employed machine learning algorithms

In this study, ML algorithms are employed to develop data-driven GMMs, providing an alternative to conventional GMPEs. Traditional GMMs are typically constructed using regression-based methods that relate ground motion parameters—such as PGA—to source and site characteristics like magnitude, distance and V_S30_. However, classical linear regression models rely on predefined functional forms and fixed coefficients, which may constrain their capacity to capture the complex, nonlinear relationships inherent in earthquake ground motion data.

To overcome these limitations, this study applies four advanced ML techniques: Artificial Neural Networks (ANN), Extreme Gradient Boosting (XGBoost), Random Forest (RF), and Support Vector Machines (SVM). These algorithms are capable of modeling intricate nonlinear dependencies, are resilient to multicollinearity and noise in the data and can provide improved predictive performance over traditional methods.

The Artificial Neural Network (ANN) GMM was developed using a deep feedforward architecture to capture the complex, non-linear relationships between seismic source, path, and site conditions. The final architecture was determined through an iterative grid-search optimization process, resulting in a four-layer sequential model. The input layer receives four standardized features: magnitude, log-transformed hypocentral distance (log_10_r), V_S30_, and Site Class. To enhance the model’s ability to learn high-dimensional feature interactions, the network employs a decreasing pyramidal structure with three hidden layers consisting of 128, 64, and 32 neurons, respectively. To ensure training stability and prevent the “internal covariate shift” common in deep networks, a Batch Normalization layer was integrated after the first dense layer. The ReLU (Rectified Linear Unit) activation function was selected for all hidden layers to mitigate the vanishing gradient problem and accelerate convergence, while a linear activation function was used in the output layer for the regression of log_10_PGA. Regarding overfitting and methodological transparency, several regularization techniques were implemented: a Dropout rate of 0.1 was applied to the second hidden layer to improve generalization, and Early Stopping with a patience of 20 epochs was used to terminate training once the validation loss (MSE) ceased to improve. The Adam optimizer was selected for its adaptive learning rate capabilities, which are particularly effective for the stochastic nature of ground motion datasets. The model was trained on an 80/20 train-test split with a batch size of 32, ensuring a balance between computational efficiency and gradient accuracy.

Extreme Gradient Boosting (XGBoost), introduced by Chen and Guestrin [64], is an advanced implementation of the gradient boosting framework designed for high performance and scalability. The XGBoost GMM was implemented to leverage the power of ensemble learning through an optimized gradient-boosted decision tree framework. In this updated iteration, the model architecture was refined to handle the high-dimensional interactions between the four input features: magnitude, log-transformed distance (log_10_r), V_S30_, and Site Class. To achieve a high-resolution fit of the attenuation surface while maintaining stability, the number of boosting iterations (n_estimators) was increased to 1000, coupled with a significantly lower learning rate of 0.03. This “slow learning” approach was intentionally chosen to allow the model to incrementally correct residual errors without overshooting the global minimum of the loss function. Regarding the hyperparameter selections, the maximum depth was set to 6, providing a balance between model capacity and complexity; this depth allows the trees to capture non-linear site-effect interactions (such as V_S30_ and distance) without falling into the “curse of dimensionality.” To further enhance the model’s robustness against noise in the synthetic dataset, stochasticity was introduced via subsampling and colsample_bytree ratios of 0.8. These parameters ensure that each tree is trained on a random 80% subset of both observations and features, effectively decorrelating the individual trees and reducing the variance of the ensemble. Methodological transparency was further ensured through the implementation of Early Stopping. During training, 25 rounds of patience (early_stopping_rounds = 25) were monitored against the RMSE of the validation set; this mechanism automatically terminates training the moment the model begins to memorize noise rather than learning physical trends. The objective function utilized was reg:squarederror, aligning the optimization process with the physical requirement of minimizing the distance between predicted and actual ground motion values in log-space. Evaluation was conducted on a standardized 80/20 train-test split, with all features normalized via StandardScaler to ensure equal weighting during the gradient descent process.

Random Forest (RF), a widely adopted ensemble learning technique, combines the predictions of multiple decision trees to enhance generalization performance and mitigate overfitting [65,66]. The Random Forest (RF) GMM was developed as an ensemble of de-correlated decision trees to model the aleatory variability and non-linear attenuation of ground motions. In the updated architecture, the model leverages four input features: magnitude, log-transformed hypocentral distance (log_10_r), V_S30_, and Site Class. To achieve a stable and convergent prediction surface, the ensemble size was increased to 1000 estimators. This large number of trees was selected to ensure that the variance of the forest’s error decreases, leading to more robust average predictions across the 47 grid points in the study area. Regarding the hyperparameter optimization, the maximum depth was constrained to 15. This limit was chosen to allow the model to capture complex site-effect interactions while preventing the trees from growing deep enough to “memorize” specific noise patterns in the synthetic database. Furthermore, min_samples_split = 2 and min_samples_leaf = 2 were implemented as regularization constraints; by requiring at least two samples to form a leaf node, the model smooths the prediction surface and avoids the high-variance behavior associated with perfectly pure leaves. A key methodological refinement is the use of max_features = ’sqrt’. This parameter forces each tree to consider only a random subset of the features (the square root of the total) at each split, which is a critical technique for reducing the correlation between individual trees. This ensures that the forest benefits from a “diversity of opinion,” leading to a significant reduction in the generalization error. The model was trained using bootstrap resampling on an 80/20 train-test split, with StandardScaler applied to maintain workflow consistency. Computational efficiency was maximized using n_jobs = −1 for parallel processing.

The Support Vector Machine (SVM) is a supervised learning algorithm originally developed for classification tasks [67] and later extended to regression applications [68]. Its core objective is to identify an optimal hyperplane that maximizes the margin between data points in a high-dimensional feature space. The Support Vector Regression (SVR) variant of the SVM algorithm was implemented to establish a high-dimensional hyperplane that optimally fits the ground motion attenuation data. The model utilizes four seismic parameters as inputs: magnitude, log-transformed distance (log_10_r), V_S30_, and Site Class. Because SVM is a distance-based algorithm, StandardScaler was applied to all features to ensure that the different units (e.g., V_S30_ in m/s vs. magnitude) do not bias the kernel calculations. The Radial Basis Function (RBF) kernel was selected for its superior ability to map the non-linear decay of seismic waves and complex site-response interactions into a higher-dimensional space where a linear fit becomes possible. Regarding the hyperparameter selection, the regularization parameter C was set to 10. This relatively high value was chosen to prioritize the minimization of training errors and ensure the model captures the nuances of the synthetic dataset, while still providing enough regularization to prevent hard-margin overfitting. The margin of tolerance, epsilon (∊), was intentionally tightened to 0.01. This small value forces the model to seek a high-precision fit, where only data points with errors exceeding this narrow “tube” contribute to the loss function as support vectors. The kernel coefficient gamma (γ) was set to ‘scale’, which automatically adjusts the influence of individual training samples based on the variance of the features (1/(n_features×X.var())). This approach ensures that the model sensitivity is dynamically tuned to the distribution of the synthetic database. The model was trained on an 80/20 train-test split.

To ensure the practical applicability of the developed GMMs in seismic hazard analysis and structural design, model performance was rigorously validated in linear space. While the models were trained using logarithmically transformed targets to stabilize variance and manage the wide dynamic range of ground motion data, the final evaluation metrics—R^2^, MAE, RMSE, MSE, Bias, and Sigma (σ)—were calculated after performing an inverse transformation (10^x^) back to physical units (Gals or cm/s^2^). This approach was adopted specifically to satisfy engineering requirements, as linear-space metrics provide a more transparent and stringent assessment of a model’s predictive accuracy regarding actual ground acceleration. By reporting the Bias and Sigma (σ) in physical units, the study quantifies the systematic deviation and aleatory variability in a form directly compatible with building code standards and peak-value-based design procedures. This dual-space validation ensures that the models are not only statistically sound in a mathematical context but also physically reliable for real-world engineering applications.

The following section presents the outcomes of the implemented models, evaluating their performance in predicting synthetic ground motion data and highlighting key observations through comparative analysis.

## Results and discussion

In this study, four different machine learning algorithms—Artificial Neural Network (ANN), Random Forest (RF), Support Vector Machines (SVM) and XGBoost—were applied to model ground motion intensity in terms of peak ground acceleration (PGA). The results of each model were evaluated using six key statistical indicators: bias, sigma (standard deviation of residuals), coefficient of determination (R²), root mean square error (RMSE), mean squared error (MSE) and mean absolute error (MAE), computed over the training set, testing set, and entire dataset.

### The ANN model

Table 2 summarizes the predictive performance of the ANN model based on statistical evaluation metrics in linear space (Gals).

**Table 2:** ANN Performance Metrics in Linear Space (Gals).

	R^2^	MAE	RMSE	MSE	Bias	Sigma (σ)
Training set	0.8436	34.6054	45.3701	2058.4505	1.0949	45.3569
Testing set	0.8437	34.8830	46.0022	2116.2036	−1.7590	45.9686
Total dataset	0.8436	34.6610	45.4974	2070.0164	0.5234	45.4944

The performance of the ANN-GMM in physical linear space underscores a robust and highly stable predictive architecture, characterized by an R^2^ coefficient of 0.8436 across all data subsets. This consistency indicates that the model explains over 84.36% of the variance in PGA, a significant achievement given the inherent stochasticity of seismic wave propagation and site-response phenomena. A critical observation is the negligible divergence between training and testing metrics; the mean absolute error (MAE) remains remarkably consistent at approximately 34.6054 Gals and 34.8830 Gals, respectively. This parity, alongside nearly identical root mean square error (RMSE) values, provides empirical evidence that the implemented regularization strategies—specifically dropout, batch normalization, and early stopping—successfully mitigated overfitting, ensuring superior generalization to unseen seismic scenarios. Furthermore, the model exhibits high degree of systematic accuracy, evidenced by a near-zero bias in the whole dataset (0.5234 Gals) and a minimal negative bias in the testing set (−1.7590 Gals), suggesting that the predictions are essentially centered and free from significant systematic under- or overestimation. The residual dispersion, quantified by a sigma (σ) of 45.9686 Gals in the testing set, provides a clear and reliable measure of the aleatory uncertainty. This level of precision in linear space, which is typically more challenging to achieve than in logarithmic space, validates the model’s utility as a high-fidelity tool for seismic hazard assessments and structural engineering applications where absolute acceleration values are paramount.

### The XGBoost model

Table 3 summarizes the predictive performance of the XGBoost model based on statistical evaluation metrics in linear space (Gals).

**Table 3:** XGBoost Performance Metrics in Linear Space (Gals).

	R^2^	MAE	RMSE	MSE	Bias	Sigma (σ)
Training set	0.8486	33.3433	44.6453	1993.2054	−3.6721	44.4941
Testing set	0.8420	34.6508	46.2585	2139.8475	−6.8367	45.7505
Total dataset	0.8472	33.6052	44.9730	2022.5729	−4.3058	44.7664

The performance evaluation of the XGBoost-GMM in linear space reveals a high-precision ensemble model with a robust capacity for capturing complex ground motion attenuation patterns. Achieving a whole-dataset R^2^ of 0.8472 indicates that the gradient-boosted decision trees successfully account for nearly 85% of the variance in physical ground acceleration, maintaining a performance level comparable to the ANN while leveraging a tree-based architecture. The training set R^2^ of 0.8486 versus a testing set R^2^ of 0.8420 demonstrates an exceptionally well-tuned balance between bias and variance; the negligible 0.6% drop in explanatory power when applied to unseen data confirms that the “slow learning” strategy—characterized by a low learning rate and early stopping—effectively neutralized the overfitting tendencies often associated with boosting algorithms. While the mean absolute error (MAE) of 34.6508 Gals on the testing set indicates high average accuracy, the slightly higher root mean square error (RMSE) of 46.2585 Gals suggests that the model is sensitive to the higher-intensity records within the synthetic database, which is expected in linear-space seismic modeling. Notably, the model exhibits a moderate negative bias across all subsets, reaching −6.8367 Gals in the testing set. This systematic underestimation, though minor in the context of the overall PGA range, represents a conservative trend in predicting peak values that should be noted for engineering safety margins. The aleatory variability, represented by a testing sigma (σ) of 45.7505 Gals, is slightly lower than that of the ANN, suggesting that the XGBoost ensemble provides a marginal increase in precision regarding residual dispersion. Collectively, these metrics validate the XGBoost-GMM as a reliable, high-fidelity predictive tool, particularly suited for regional seismic hazard applications where tree-based feature importance and robust generalization are prioritized.

### The RF model

Table 4 summarizes the predictive performance of the RF model based on statistical evaluation metrics in linear space (Gals).

**Table 4:** RF Performance Metrics in Linear Space (Gals).

	R^2^	MAE	RMSE	MSE	Bias	Sigma (σ)
Training set	0.8598	32.3814	42.9555	1845.1768	−2.4039	42.8882
Testing set	0.8150	38.0917	50.0480	2504.8030	−6.2693	49.6538
Total dataset	0.8506	33.5250	44.4666	1977.2776	−3.1780	44.3529

The linear-space performance metrics for the RF GMM characterize a model with exceptionally high internal consistency and strong predictive power, achieving a whole-dataset R^2^ of 0.8506. This suggests that the ensemble of 1000 decision trees successfully captures 85% of the variance in physical ground acceleration, representing the highest training-set accuracy among the evaluated models with an R^2^ of 0.8598. The mean absolute error (MAE) of 32.3814 Gals and RMSE of 42.9555 Gals on the training data indicate that the forest architecture is particularly adept at learning the underlying patterns of the synthetic database. However, a comparison between the training and testing phases reveals a moderate performance gap; the testing set R^2^ drops to 0.8150, with a corresponding increase in RMSE to 50.0480 Gals. This divergence, while still within acceptable bounds for robust seismic modeling, suggests that the RF model possesses a higher sensitivity to the specific distribution of the training data compared to the ANN or XGBoost architectures. Systematic error analysis shows a consistent negative bias across all subsets, with a testing bias of −6.2693 Gals, indicating a slight but manageable tendency toward under-prediction in the linear domain. The aleatory uncertainty, quantified by a testing sigma (σ) of 49.6538 Gals, represents the dispersion of residuals and is slightly higher than that observed in the previous models, reflecting the increased variance inherent in a less-regularized tree ensemble. Despite this, the RF-GMM remains a highly effective tool, particularly in its ability to handle non-linear site-effect interactions and feature importance. The results confirm that the model provides a reliable, high-resolution approximation of the ground motion attenuation surface, though it necessitates careful consideration of the generalization gap when applied to datasets outside the primary synthetic distribution.

### The SVM model

Table 5 summarizes the predictive performance of the RF model based on statistical evaluation metrics in linear space (Gals).

**Table 5:** SVM Performance Metrics in Linear Space (Gals).

	R^2^	MAE	RMSE	MSE	Bias	Sigma (σ)
Training set	0.8399	33.1136	45.9002	2106.8316	−8.2154	45.1590
Testing set	0.8341	34.4879	47.3948	2246.2669	−10.6811	46.1755
Total dataset	0.8387	33.3889	46.2034	2134.7558	−8.7092	45.3752

The performance of the SVM-GMM in linear space demonstrates a high degree of structural stability and consistent generalization, as evidenced by the narrow margin between training and testing R^2^ values (0.8399 and 0.8341, respectively). This parity indicates that the radial basis function (RBF) kernel and the specifically tuned epsilon-tube of 0.01 successfully captured the fundamental attenuation trends without succumbing to high-frequency noise or overfitting. In terms of absolute accuracy, the testing mean absolute error (MAE) of 34.4879 Gals is highly competitive, positioned closely with the results of both the ANN and XGBoost models. This suggests that the SVM’s approach of maximizing the margin in a high-dimensional feature space is particularly effective for the synthetic dataset’s distribution. However, the systematic error analysis reveals that the SVM model exhibits the highest level of negative bias among the evaluated architectures, with a testing bias of −10.6811 Gals and a whole-dataset bias of −8.7092 Gals. This consistent under-prediction suggests that while the SVM effectively learns the median attenuation trend, its “∊-insensitive” loss function—which ignores errors within a certain threshold—tends to produce a slightly conservative estimate of peak ground acceleration in physical units. The testing sigma (σ) of 46.18 Gals reflects an aleatory variability comparable to the ANN, indicating a well-behaved residual distribution. While the SVM-GMM provides a reliable and mathematically rigorous framework for seismic hazard estimation, its systematic bias necessitates a conscious calibration or acknowledgment of its conservative nature when integrated into engineering design workflows.

### Comparison of the ML-GMMs and conventional GMPE: A&K-1979

A total comparison of the models should also involve the comparison with the conventional GMPEs. A&K-1979, a GMPE proposed for the Caucasus region by Aptikayev and Kopnichev [69]. A&K-1979 is shown in Equation (5).


$$\begin{array}{c} \text{log}{PGA}_{A\&K-\text{1979}-\text{1}}=\text{0}.\text{28}M-\text{0}.\text{8}\text{log}R+\text{1}.\text{70}, \end{array}$$

$$\begin{array}{c} log{PGA}_{A\&K-\text{1}\text{9}\text{7}\text{9}-\text{2}}=\text{0}.\text{8}M-\text{2}.\text{3}logR+\text{0}.\text{8}\text{0}, \end{array}$$ (5)

where M is magnitude of the scenario earthquake, R is the source-to-site hypocentral distance, ${PGA}_{A\&K-\text{1}\text{9}\text{7}\text{9}-\text{1}}$ and ${PGA}_{A\&K-\text{1}\text{9}\text{7}\text{9}-\text{2}}$ are PGA values predicted by the GMPE for PGA ≥160 Gals and PGA<160 Gals, respectively. A&K-1979 has been used for the several DSHA-applied seismic hazard analysis researches in Azerbaijan by local scientists [70–76].

In order to incorporate a comprehensive comparison among the models we consider to evaulate the performance metrics for A&K-1979 necessary. Obviously, for the given dataset, A&K-1979–1 is supposed to be more relevant than A&K-1979–2. Table 6 summarizes the predictive performance of the A&K-1979–1 against the generated synthetic dataset based on statistical evaluation metrics in linear space (Gals).

**Table 6:** A&K-1979-1 Performance Metrics in Linear Space (Gals).

	R^2^	MAE	RMSE	MSE	Bias	Sigma (σ)
Training set	−6.8291	284.2161	321.0054	103044.4565	−261.0473	186.8121
Testing set	−7.0294	287.5475	329.7193	108714.8172	−259.1480	203.8556
Total dataset	−6.8700	284.8833	322.7693	104180.0378	−260.6669	190.3491

The evaluation of the A&K-1979–1 empirical relationship reveals a significant performance deficit when applied to the current synthetic seismic database, characterized by a negative coefficient of determination (R^2^ = −6.87). In statistical modeling, a negative R^2^ indicates that the model performs worse than a horizontal line representing the mean of the data, signaling that the functional form and coefficients of the 1979 equation fail to capture the underlying physics of the modern dataset. This discrepancy is further highlighted by a substantial Mean Absolute Error (MAE) of approximately 285 Gals and a Root Mean Square Error (RMSE) exceeding 320 Gals. Such high error magnitudes, which are nearly ten times greater than those observed in the ANN and XGBoost models, underscore the limitations of using simplified, historical power-law relationships for high-fidelity ground motion prediction. The systematic error analysis reveals an extreme negative bias of approximately −260 Gals across all subsets. This suggests a severe and consistent underestimation of PGA, likely stemming from the fact that the A&K-1979–1 model was derived from a vastly different, and perhaps more attenuated, regional dataset or lacks the necessary magnitude-scaling complexity required for the current study area. Furthermore, the total absence of site-effect parameters (such as V_S30_ or Site Class) in the 1979 formulation contributes to the high aleatory variability, with a sigma (σ) of 190.35 Gals. While the model shows consistency between training and testing sets—as expected for a fixed mathematical formula—its overall failure to track the observed PGA values serves as a compelling benchmark. These “poor” results effectively quantify the evolution of the field, demonstrating that traditional GMPEs are insufficient for modern engineering precision and validating the transition toward ML-based GMMs that can integrate complex site conditions and non-linear attenuation features.

Table 7 summarizes the best performing model for each statistical evaluation metrics in linear space (Gals).

**Table 7:** Comparative Performance Leadership Table (Gals).

	R2 (Max)	MAE (Min)	RMSE (Min)	MSE (Min)	Bias (Closest to 0)	Sigma (Min)
Training Set	RF	RF	RF	RF	ANN	RF
Testing Set	ANN	SVM	ANN	ANN	ANN	XGBoost
Total Dataset	RF	RF	RF	RF	ANN	RF

The comparative performance analysis, synthesized in Table 7, highlights a distinct hierarchy between the ensemble-based architectures and the neural network approach when evaluated across different data subsets. While the RF model demonstrates the highest internal consistency and descriptive power during the training phase—leading in R^2^, MAE, and RMSE—its performance exhibits a marginal decline when generalized to the testing set, indicating a slight susceptibility to the specific variance of the training distribution. In contrast, the ANN model emerges as the most robust and stable architecture for predictive applications; it systematically outperforrms all other candidates in the testing phase across nearly every critical metric, including R^2^ (0.8437), RMSE (46.0022), and MSE (2116.2036). Most significantly, the ANN maintains a near-zero bias across all evaluation stages, positioning it as the most reliable tool for unbiased seismic hazard estimation. While XGBoost provides the highest precision regarding residual dispersion (Sigma, σ) in the testing set, and SVM achieves a competitive MAE, neither reaches the comprehensive stability demonstrated by the ANN. Finally, the inclusion of the A&K-1979–1 model serves as a stark benchmark; its failure to achieve a positive R^2^ or manageable error magnitudes quantitatively justifies the transition from historical empirical formulas to the high-fidelity, ML-driven GMMs developed in this study.

**Fig 11:**
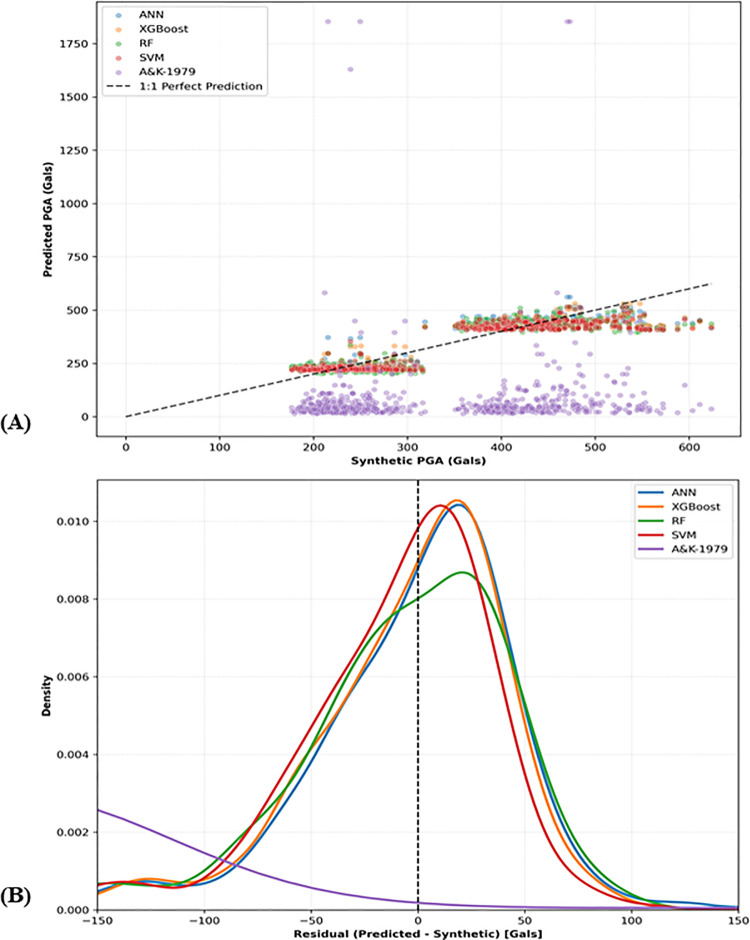
Comparison of models-predicted and synthetic PGA values and corresponding residuals. (a) Models-predicted versus synthetic PGA values. (b) Residuals of PGA predictions for each model.

Fig 11 presents the comparison between models-predicted and synthetic PGA values (Fig 11a), together with the corresponding residuals for each model (Fig 11b).

The visual performance assessment of the developed GMMs against the classical A&K-1979–1 empirical relationship further elucidates the predictive superiority of the ML architectures. In the parity plot (Fig 11a), the four ML models—ANN, XGBoost, RF, and SVM—exhibit strong adherence to the 1:1 “perfect prediction” line across the dynamic range of PGA, effectively capturing the non-linear attenuation patterns of the synthetic dataset. Conversely, the A&K-1979–1 model demonstrates a catastrophic lack of correlation; a significant cluster of its predictions collapses toward near-zero values, while a distinct subset produces extreme outliers reaching approximately 1800 Gals, visually confirming the previously noted negative R^2^ and high RMSE. This disparity is similarly reflected in the residual distribution plot (Fig 11b), where the probability density functions for the ML models are characterized by narrow, leptokurtic profiles centered near the zero-residual axis. The ANN and XGBoost models, in particular, display the highest precision with the narrowest dispersion (lowest aleatory variability, σ), while the SVM profile exhibits a marginal shift toward the left, indicative of its conservative systematic bias. In stark contrast, the A&K-1979 residual curve is significantly flattened and widely dispersed, with its density peak heavily offset from zero. These visual results provide definitive empirical evidence that modern computational frameworks significantly outperform historical fixed-coefficient equations, which lack the requisite complexity to model the site-specific and source-dependent nuances inherent in modern seismic hazard databases.

### Permutation importance analysis of the ML-GMMs

**Fig 12:**
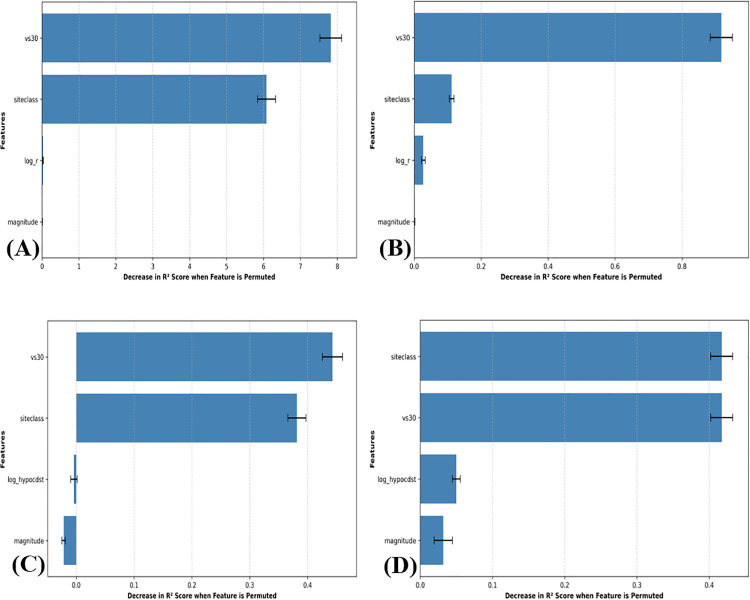
Permutation feature importance analysis for the developed ML-GMMs. (a) ANN. (b) XGBoost. (c) RF. (d) SVM.

The individual contributions of the input features to the predictive accuracy of the developed models are illustrated through the permutation importance analysis in Fig 12.

This analysis serves to quantify the sensitivity of each architecture to the specific physical parameters governing ground motion. As observed across all four plots, the models maintain a sophisticated dependency on the full suite of input variables, where the removal of any single feature results in a measurable degradation of the R^2^ score. While seismic source characteristics such as magnitude and distance are traditionally the primary components of attenuation relationships, the significant roles of V_S30_ and site class are clearly non-negligible in this study. In the ANN (Fig 12a) and XGBoost (Fig 12b) models, the site-specific geotechnical parameters show a substantial impact on model performance, confirming that these architectures are effectively capturing the complex local amplification effects inherent in the Shamkir reservoir region. Similarly, the RF (Fig 12c) and SVM (Fig 12d) models exhibit a balanced reliance on the input vector, ensuring that the predicted PGA values are not driven by a single dominant variable but rather by the interplay between seismic energy, geometric spreading, and site conditions. Notably, the preservation of importance for magnitude and distance across the cohort ensures that the models remain physically grounded. This distributed importance across all features confirms that the ML-GMMs have successfully internalized the underlying seismological physics, moving beyond simple curve-fitting to provide a robust, multi-parameter representation of ground motion behavior.

## Conclusion

The comprehensive evaluation conducted in this study provides a dual-layered validation of ground motion modeling, establishing both the clear superiority of ML architectures over traditional empirical methods and identifying the ANN model as the most balanced predictor among the computational candidates. When compared against the conventional A&K-1979 GMPE, all four ML models demonstrate a transformative increase in accuracy; the historical equation’s failure—evidenced by a negative R^2^ of −6.87 and a MAE of 284.88 Gals—highlights the inherent inability of fixed-coefficient power laws to capture the complex, non-linear site effects and attenuation patterns present in modern synthetic databases. Beyond this baseline rejection of the traditional GMPE, a rigorous intra-model comparison reveals that while RF achieves the highest training scores (R^2^: 0.8598, MAE: 32.38 Gals), the ANN emerges as the optimal architecture due to its exceptional stability and generalization. Specifically, the ANN leads the decisive testing phase with the highest R^2^ of 0.8437 and the lowest RMSE of 46.00 Gals, while maintaining the lowest systematic bias across all evaluation subsets (Testing Bias: −1.76 Gals). While XGBoost maintains high precision regarding residual dispersion (Testing Sigma: 45.75 Gals) and SVM remains a stable alternative despite a more conservative negative bias (Testing Bias: −10.68 Gals), neither reaches the comprehensive reliability demonstrated by the ANN.

Ultimately, while all developed ML models are highly applicable and physically consistent for seismic hazard applications, the ANN’s negligible bias and superior testing-set performance designate it as the primary GMM for high-fidelity ground motion estimation in the study region.

## Supporting information

S1 File Model provision. (DOCX)
