# Supplementary material for: Machine learning-based ground motion simulation for seismic hazard assessment of critical water infrastructure in Azerbaijan (Case study: Major Shamkir water reservoir)
Source: PLoS One. 2026 Apr 1;21(4):e0344984. doi: 10.1371/journal.pone.0344984 (PMC13043061; doi:10.1371/journal.pone.0344984)
Supplement: S1 File — (DOCX) [file pone.0344984.s001.docx]

**Model provision**

We provide access to the developed Ground Motion Models (GMMs) through our GitHub repository. The repository includes the trained models for ANN (in .h5 format), XGBoost (in .json format), RF (in .pkl format) and SVM (in .pkl format) along with their corresponding scaler files (in .pkl format) used for input normalization. Additionally, it contains a Jupyter Notebook (PONE_GMMs_Load_Predict.ipynb) with scripts to load each model and perform PGA predictions for magnitude, hypocentral distance, V_S30_ values and site class. A README file is also provided to guide users through the setup and usage process.

The GitHub repository is available at: <https://github.com/turalbabaev/PONE_Manuscript_2026>.
